# Fabrication of Hollow Acrylic Vaginal Stents Using Frozen Coconut Oil for Vaginal Agenesis Management

**DOI:** 10.7759/cureus.60512

**Published:** 2024-05-17

**Authors:** Jahnavi P Gorripati, Surekha A Dubey, Sharayu Nimonkar, Madhu Priya

**Affiliations:** 1 Department of Prosthodontics, Sharad Pawar Dental College, Datta Meghe Institute of Medical Sciences, (Deemed to be University), Wardha, IND

**Keywords:** rehabilitation, prosthetic, maxillofacial prosthesis, interdisciplinary, hollow vaginal stent

## Abstract

In a rare condition known as vaginal agenesis, the uterus (womb) may develop only partially or not at all, while the vagina fails to develop altogether. It is common to diagnose vaginal agenesis, when a female does not start menstruation at puberty. This is a prenatal disorder that may also be linked to bone or kidney issues. Mullerian agenesis, Mullerian aplasia, and Mayer-Rokitansky-Kuster-Hauser (MRKH) syndrome are other names for the illness. Treatment modalities encompass surgical and nonsurgical interventions, including the utilization of prefabricated or customized vaginal stents for neovagina reconstruction and maintenance. This case report describes the development of a neovagina in a 27-year-old female diagnosed with vaginal agenesis, a characteristic of MRKH syndrome. A customized clear acrylic stent, designed to provide a resilient surface, serves as a straightforward and cost-efficient alternative for managing this condition. Significantly, it enhances patient's compliance and comfort during treatment, addressing both the physical and psychological ramifications of this congenital anomaly. This customized vaginal stent not only provides a practical solution but also contributes to enhancing the quality of life for individuals grappling with vaginal agenesis, thereby offering a promising avenue for addressing the multifaceted challenges associated with this condition.

## Introduction

Mayer-Rokitansky-Kuster-Hauser (MRKH) syndrome, alternatively referred to as Mullerian agenesis or congenital absence of the uterus and vagina (CAUV), is a congenital condition marked by the partial or complete lack of the uterus and the upper portion of vagina in individuals assigned female at birth. This condition typically manifests as primary amenorrhea during adolescence due to absence of menstruation [1]. MRKH syndrome affects approximately 1 in 4,500 female births and is believed to result from abnormal development of the Mullerian ducts during embryonic development. While the exact cause remains unclear, MRKH syndrome is thought to involve both genetic and environmental factors. It is often associated with other congenital anomalies, such as renal, skeletal, or hearing abnormalities. Treatment options for MRKH syndrome include both surgical and nonsurgical approaches, aimed at addressing reproductive and psychological needs, to achieve neovagina creation and improve the quality of life for affected individuals [2].

The Abbe-McIndoe technique, named after surgeons Robert Abbe and Harold McIndoe, is a surgical procedure used for the creation of neovagina in individuals with congenital vaginal agenesis or other conditions leading to vaginal underdevelopment with a success rate of 83-96% [3,4]. This technique involves the surgical formation of a vaginal canal using a skin graft or other tissue, typically from the patient's own body, to construct a functional vagina. During the Abbe-McIndoe procedure, a space for the neovagina is created between the bladder and rectum. A skin graft, often taken from the inner thigh or buttocks, is then fashioned into a tube and placed into this space to form the new vaginal canal. The graft is sutured in place and left to heal, allowing the development of a functional vaginal passage. This article describes a straightforward method for making a custom hollow acrylic vaginal stent for a young female patient with MRKH syndrome who underwent surgical neovaginal canal creation utilizing the Abbe-McIndoe procedure [5].

## Case presentation

The department of prosthodontics received a referral for a 27-year-old female patient with primary amenorrhea. Despite displaying typical secondary sexual characteristics, a gynecological examination revealed vaginal agenesis, prompting a recommendation for ultrasound imaging. Counseling about benefits related to the surgical procedure and post-surgical stent use regularly to the patient and family members.

**Figure 1:**
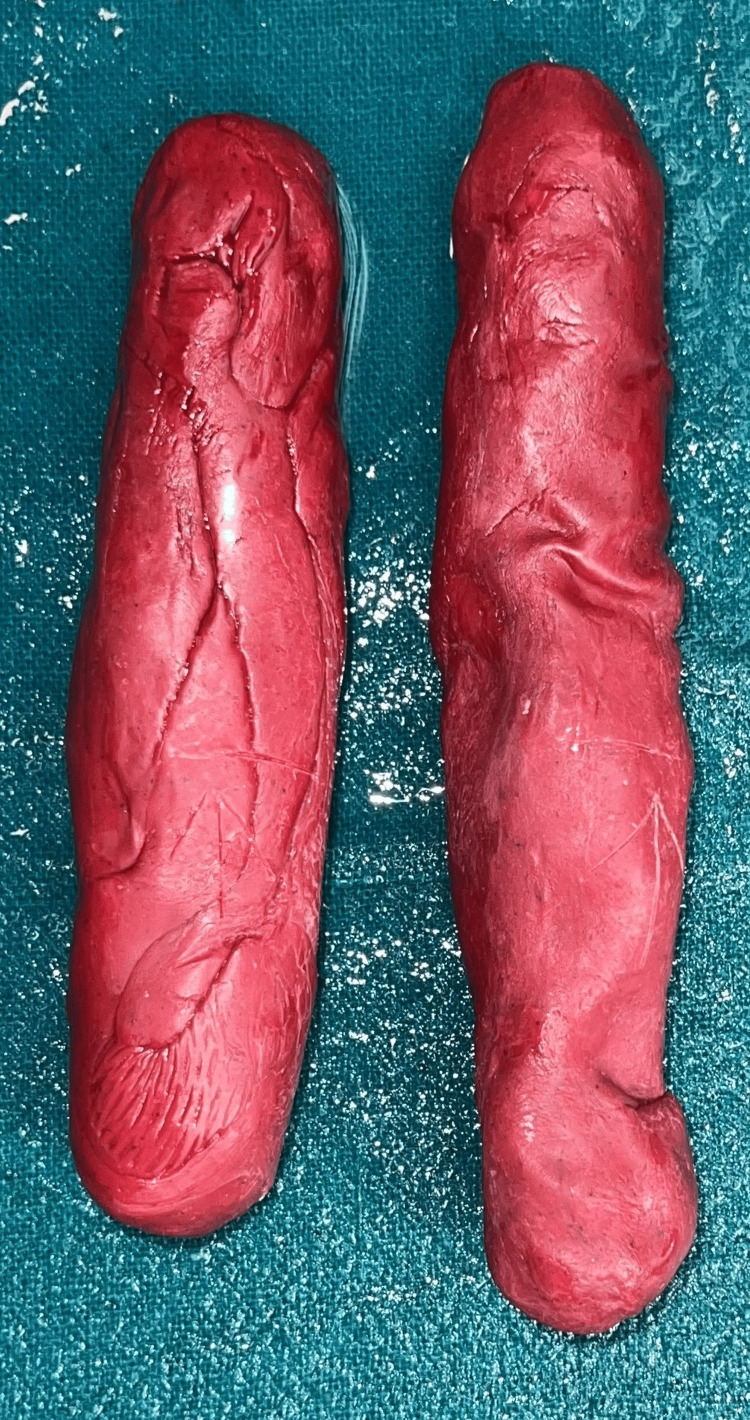
Impression making using impression compound

**Figure 2:**
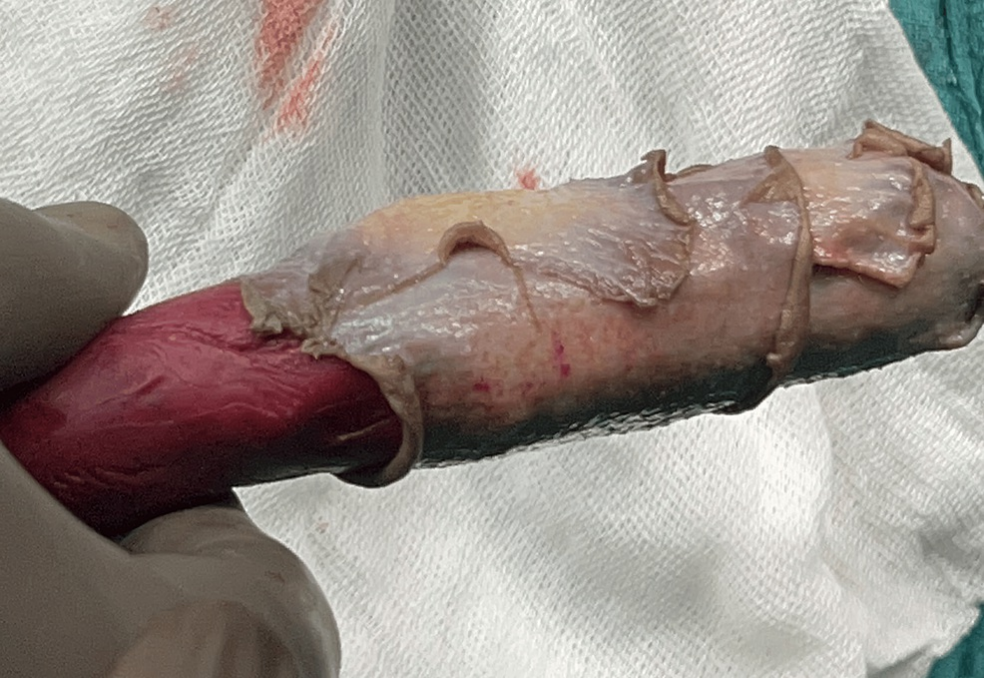
Subcutaneous graft over the impression.

One possible direction of treatment for the patient was to create neovagina with the technique of Abbe McIndoe's operation. Vaginoplasty, addressing vaginal agenesis, was performed under general anesthesia by surgeons in the obstetrics and gynecology department. The neovagina was formed after the dissection was completed, and hemostasis was achieved. Two impressions were made for the neovagina using MDM Y-Dent's impression compound. One impression was used to fabricate the vaginal stent (Figure 1). The other impression was used as a temporary stent, around which a subcutaneous graft (obtained from a tight region) was secured with sutures. With the graft, this temporary stent was inserted into the neovagina and left in place for one week (Figure 2).

Stent preparation

**Figure 3:**
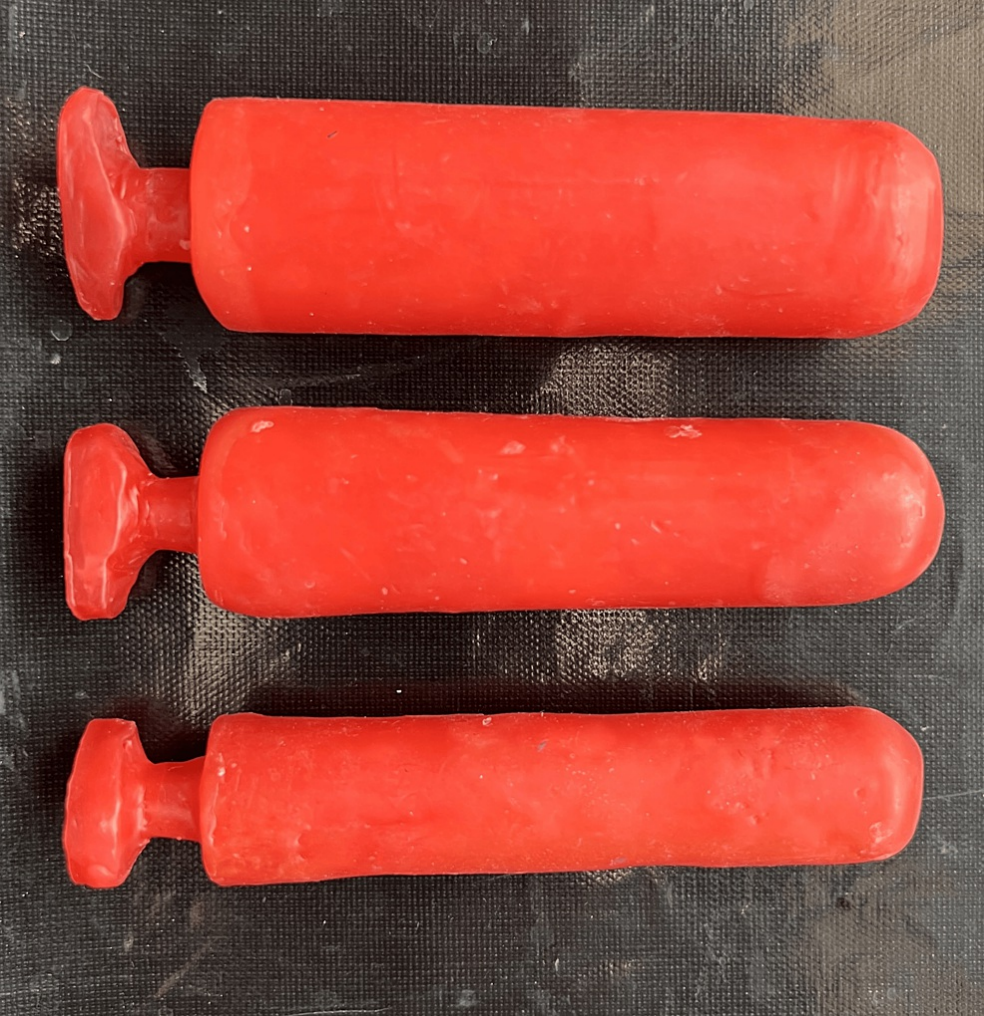
Wax patterns of the initial stent along with second and third stent

**Figure 4:**
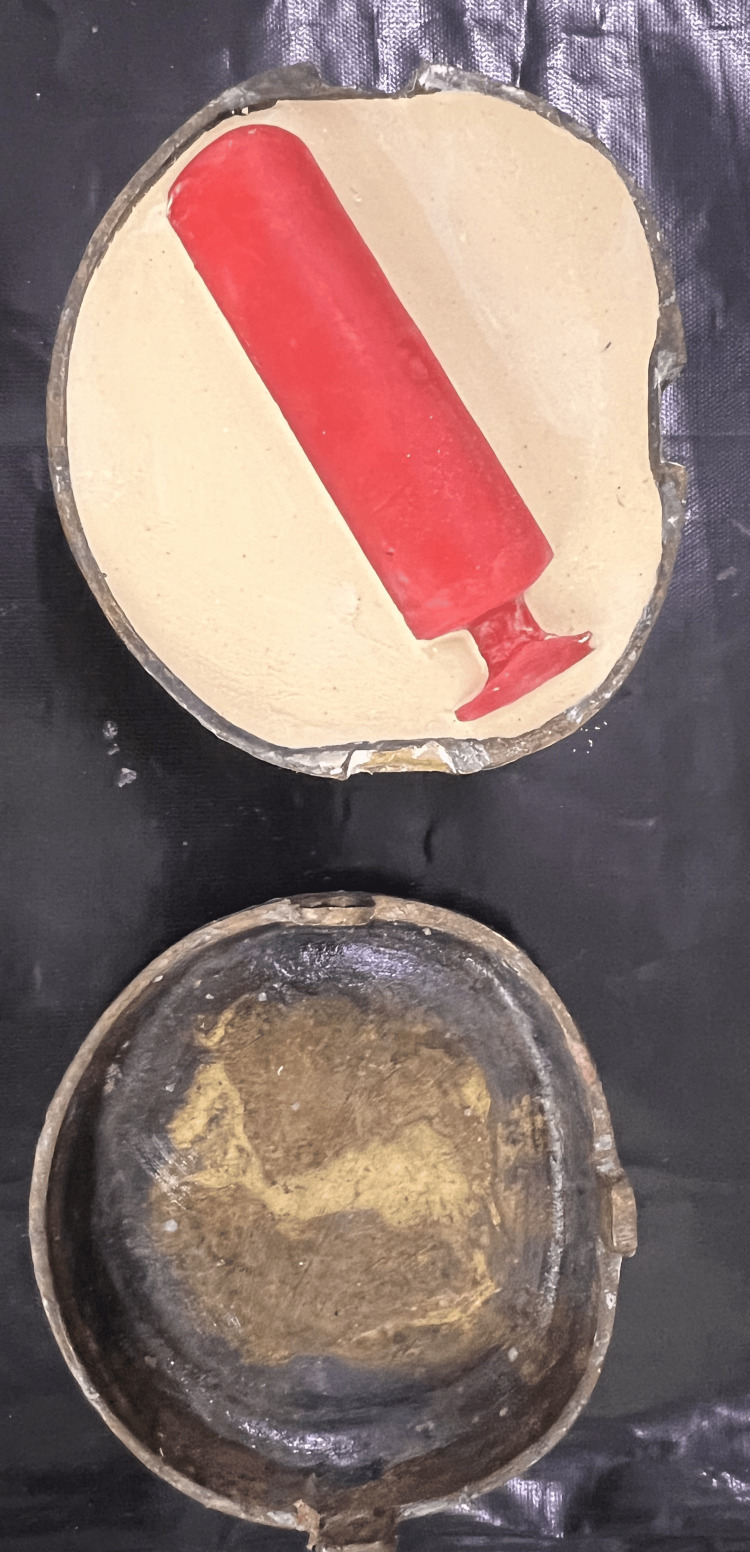
Investing of wax pattern of initial stent in dental stone

The process begins with obtaining an impression of the neovagina to capture the necessary details. This impression is then used to fabricate a cast or mold using type III dental stone (Gold Stone Dental Stone - Yellow). Subsequently, a wax pattern is meticulously crafted for the initial stent. The modeling wax pattern (2GM Modelling Dental Wax - Red), measuring 5 × 2.5 cm, equipped with a handle, is carefully verified against the cast to ensure accuracy (Figure 3). Finally, the process involves flasking, which consists of investing the wax pattern in a flask for further procedures (Figure 4).

**Figure 5:**
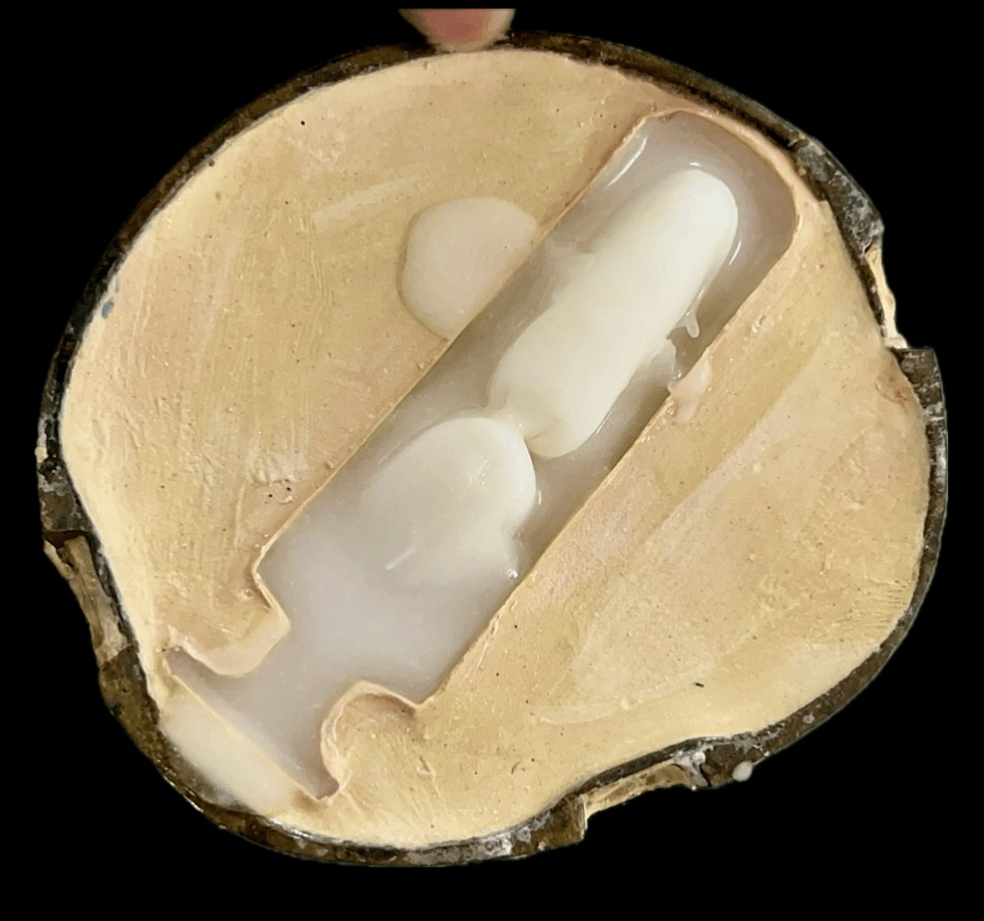
Packing of clear heat cure-acrylic resin with frozen coconut oil.

**Figure 6:**
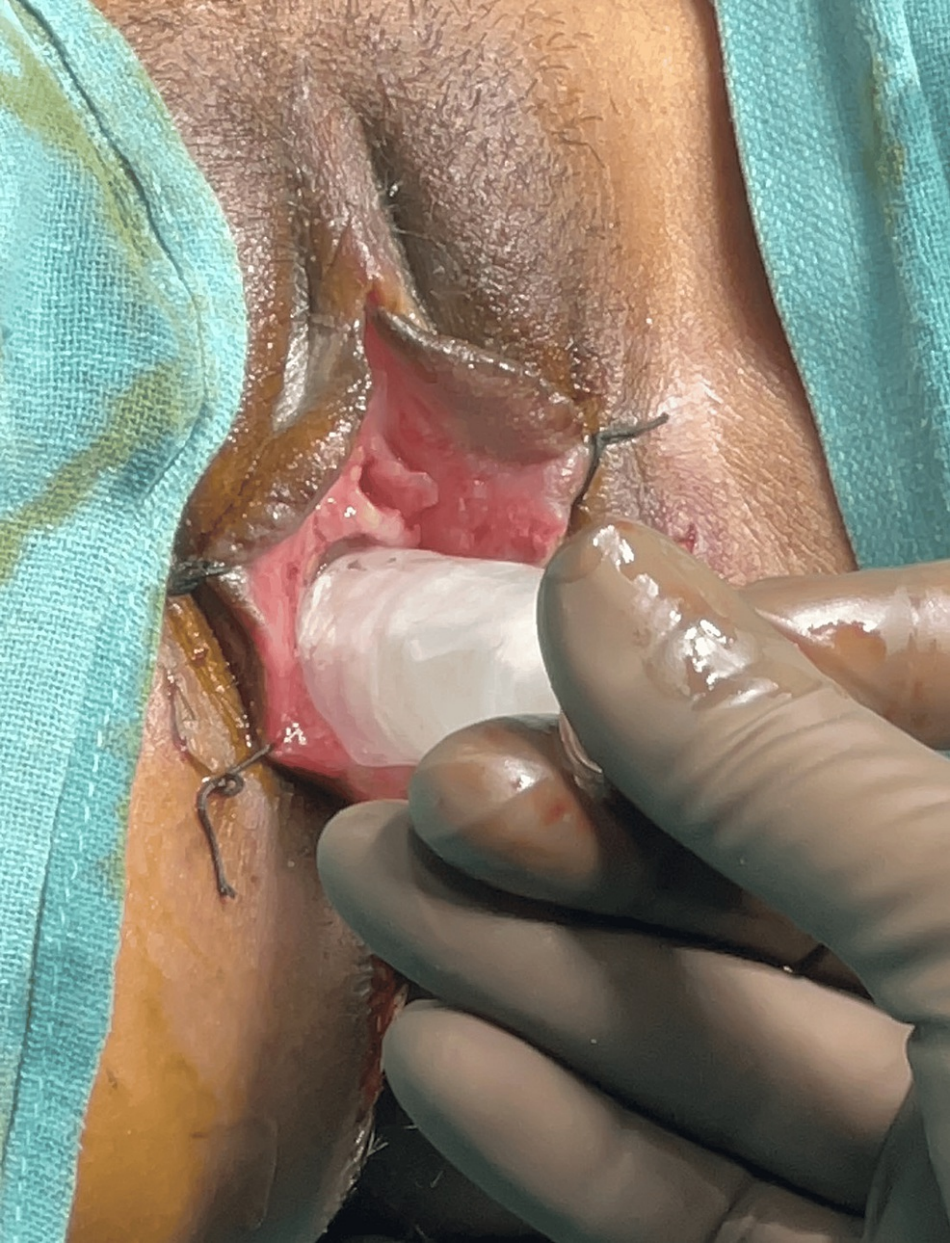
Insertion of initial stent one-week post-surgery

A second wax pattern duplicate was created utilizing a mold from frozen coconut oil, allowing a hollow stent to be fabricated in the exact shape of the first wax template. Applying separating media to both sides of the flask after dewaxing, the stent was constructed by embedding it between two layers of clear heat-cure acrylic resin and hollowing it out with frozen coconut oil during the packing process (Figure 5). After mixing and filling the two parts of the mold with heat-cure acrylic resin, the flasks were sealed, and brief curing cycles were finished. In between the two halves of the mold, a cylindrical frozen coconut oil block was positioned. The vaginal stent was carefully positioned in the neovagina to ensure a proper fit (Figure 6). To detect any bleeding, discharge, correct stent placement, or possible difficulties with sutures in the uvular region, routine monitoring was carried out. Although initially uncomfortable, the stent proved effective and gradually became acceptable to the patient.

**Figure 7:**
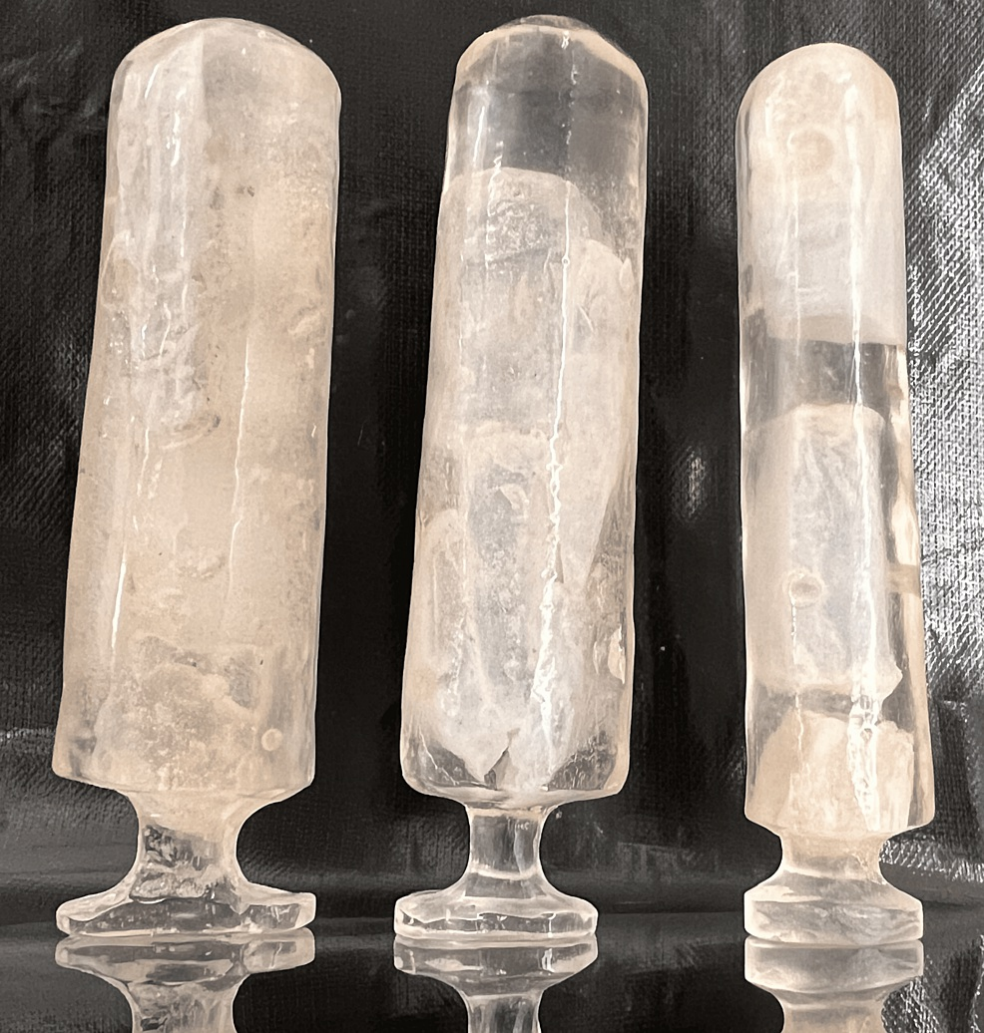
Final vaginal clear heat cure acrylic stents

To produce a hollow stent, we crafted a second wax pattern with slightly smaller dimensions than the stent. This pattern was then duplicated to create a mold with predetermined dimensions for solid coconut oil. A 2-3 mm coating of heated-cured clear acrylic resin was applied after smoothing the wax surface. The stent was made hollow using frozen coconut oil, and after solidification, it was carefully finished and polished (Figure 7). Since heat-cured acrylic reduces the percentage of monomer content and is simpler, more affordable, stronger, more resistant to wear, easier to maintain, and less prone to vaginal irritation, it was the material of choice.

A week following post-surgery, the labia sutures were taken out and the vaginal cast was carefully taken off. Follow-up visits occurred every two weeks for six months, during which the patient received instructions on maintaining the acrylic stent Dilation should occur twice every day. Early morning and right before bedtime are the recommended times. This allows you to focus on your regular activities without having to take breaks for dilatation. Initially used overnight for the first three months, the patient transitioned to continuous usage for the next three months.

## Discussion

First identified in 1961, MRKH syndrome often leads to absence of vaginal development which is a major congenital anomaly that affects the female reproductive system [1]. Despite maintaining typical female genetic, physical, and hormonal characteristics, individuals with this syndrome commonly experience abnormalities in the renal and skeletal systems. Surgical vaginal stents are frequently employed post McIndoe vaginoplasty to aid in postoperative care. This condition poses substantial challenges, affecting psychological, reproductive, and social aspects for both the individual and their parents. In certain cultural contexts, surgical correction of genital anomalies is regarded as culturally sensitive. It is important to emphasize the importance of these stents in maintaining the width and depth of the vagina, preventing stricture, and shrinking while managing bleeding. Although in the past soft molds were employed, the latest guidelines support the use of hard stents for improved results [6].

It is not generally acknowledged as a standard procedure to create a functional vagina in those who have underdeveloped or congenital vaginal absence [7]. One way to cure vaginal agenesis is to use the present mucous membrane-lined canal for neovagina, lining the surgical cavity with an autologous graft from the buccal mucosa or an allograft, like amnion [8]. The best and most recommended technique for this is still the 1888-introduced Abbe McIndoe approach [1]. Even if it is inconvenient, patient commitment to stent wear is essential since non-compliance can greatly contribute to treatment failure [9]. Vaginal stents have occasionally been replaced with a vacuum-assisted closure device, which minimizes graft rejection during vaginal reconstruction and does away with the necessity for them. Prefabricated vaginal dilators are employed in non-surgical procedures, although their effectiveness has been restricted. These methods frequently result in discomfort and significantly depend on patient's motivation [10].

Vaginal stents are frequently used postoperatively to preserve neovaginal dimensions, avoiding shrinking and contraction, and facilitating hemostasis. A systematic study has validated the advantages of stents or dilators in improving the health of females who have already experienced stenosis [11]. To maintain neovaginal dimensions, several prefabricated or customized stent types have been described. These include the silicone stent, solid or hollow acrylic stents, tissue expanders, syringes, vacuum expandable molds [12], ORFIT vaginal stent, and a novel silicone-coated acrylic stent [13] that combines both materials.

 In contrast to vaginal agenesis cases, which occur in females, male patients with gender dysphoria who undergo the creation of artificial vaginal tracts are more susceptible to vaginal wall collapse. A stent made of acrylic resin is used to prevent this because its strength and hardness are necessary to maintain patency [14]. Despite being utilized, silicone stents can deteriorate and become infected with fungi if they are not properly maintained.

A vaginal stent was made hollow to lessen the weight of the stent to increase patient compliance. Heat cure acrylic resin has various advantages for vaginoplasty procedures, including simplicity of design, ease of manufacture, cost-effectiveness, stiffness, appropriate strength, wear resistance, and less irritation. The disadvantages include high porosity, high water absorption, volume changes, and a large amount of residual monomer. The hollowing of prostheses has been made using a variety of materials, including wax, thermocol, sugar, caramel, ice, and salt [15,16]. The use of coconut oil to create the hollow area is what makes this procedure distinctive. Residues of wax may obstruct the processing of silicone and acrylic, and substances like ice, salt, sugar, caramel, and thermocol may result in an inner prosthesis surface that is abrasive and may harbor bacteria. Coconut oil, on the other hand, is inexpensive, readily accessible, less likely to cause problems, and easy to remove. A natural plant extract made from fresh coconut meat is called virgin coconut oil (VCO). VCO attracted particular interest due to its bioactive components, which are widely recognized for their antipyretic, anti-inflammatory, antibacterial, and antioxidant characteristics [17,18]. Medium-chain fatty acids, including lauric, capric, and caprylic acids, make up most of their chemical components and have been shown to have antifungal properties, especially against Candida *albicans*. Furthermore, coconut oil is a perfectly controllable and perfect option because it doesn't react with the prosthesis material.

The limitation of this technique involves the necessity of maintaining the temperature of frozen coconut oil as low as practically achievable, coupled with the requirement for using oil without added waxes.

## Conclusions

Vaginoplasty utilizing a subcutaneous graft has proven to be a successful intervention for treating vaginal agenesis. However, the adherence to postoperative stent usage significantly influences the procedure's efficacy. In this case study, the implementation of a hollow acrylic stent filled with frozen coconut oil emerges as a practical choice. This option is cost-effective, readily accessible, less likely to induce complications, and simple to remove, owing to its antipyretic, anti-inflammatory, antibacterial, and antioxidant properties. The main challenge associated with this technique lies in maintaining the temperature, to ensure the oil remains in a solid state during packing. With the primary objective of reducing soft tissue tension and enhancing the overall outcome of vaginoplasty, this method offers a straightforward, rapid, and cost-efficient approach to stent creation. Employing this collaborative and accessible technique in the management of vaginal agenesis ensures both safety and efficacy.
